# How sudden- versus slow-onset environmental events affect self-identification as an environmental migrant: Evidence from Vietnamese and Kenyan survey data

**DOI:** 10.1371/journal.pone.0297079

**Published:** 2024-01-25

**Authors:** Quynh Nguyen, Gabriele Spilker, Vally Koubi, Tobias Böhmelt

**Affiliations:** 1 Wyss Academy for Nature, Institute of Political Science, University of Bern, Bern, Switzerland; 2 Cluster of Excellence “The Politics of Inequality”, University of Konstanz, Konstanz, Germany; 3 Department of Humanities, Social and Political Sciences, Center for Comparative and International Studies, ETH Zurich, Zurich, Switzerland; 4 Department of Government, University of Essex, Colchester, United Kingdom; East China Normal University, CHINA

## Abstract

In response to changing climatic conditions, people are increasingly likely to migrate. However, individual-level survey data reveal that people mainly state economic, social, or political reasons as the main drivers for their relocation decision–not environmental motives or climate change specifically. To shed light on this discrepancy, we distinguish between sudden-onset (e.g., floods and storms) and slow-onset (e.g., droughts and salinity) climatic changes and argue that the salience of environmental conditions in individuals’ migration decisions is shaped by the type of climate event experienced. Empirically, we combine individual-level surveys with geographic information on objective climatic changes in Vietnam and Kenya. The empirical evidence suggests that sudden-onset climate events make individuals more likely to link environmental conditions to their migration decision and, hence, to identify themselves as “environmental migrants.” Regression analyses support these results and are consistent with the view that slow-onset events tend to be linked with migration decisions that are more economically motivated.

## Introduction

More frequent and more intense extreme climate events, such as storms and floods, as well as more long-term, gradual changes encompassing droughts, desertification, or sea-level rise, are expected to have far-reaching repercussions on ecosystems and humans in many countries worldwide [1]. In response to changing climatic conditions, people often choose to migrate internally, i.e., permanently move from one location in a country to another, in order to increase their chances of surviving in disaster-prone regions, to diversify their income, or, more generally, to adapt to a changing environment [2, 3]. However, there is little consensus in the existing literature regarding the direction and the extent to which climatic conditions influence internal migration [4, 5]. While some studies report that climate events contribute to increased human migration [6–9], others either point toward a null effect [10–12] or actually a decline in individuals’ relocation [13, 14]. The lack of consensus in existing work reflects the differences in the methodological approaches used regarding the conceptualization and measurement of migration and climate events, the integration and aggregation of data, and the exploration of contextual factors [15].

Having said that, recent estimates of the World Bank’s Groundswell Report suggest more than 200 million people could become internally displaced because of climate change by 2050 [16]. But this highlights an interesting puzzle that we seek to address here: when exploring individual-level survey data on migration motives, respondents mostly name economic, social, or political reasons as the key drivers behind their migration decisions, with only a few mentioning environmental conditions [17–21]. Does this imply that climatic changes are unimportant when examining migration patterns and people’s considerations about leaving their homes? In this research, we shed light on this discrepancy by analyzing the determinants of people’s self-identification as “environmental migrants.”

We focus on individuals’ exposure to objective climatic changes. Climate change manifests itself in different forms in different contexts [1]. On one hand, it is linked to a higher risk of sudden-onset and short-term events, such as storms or floods, which typically cause immediate and often immense destruction [14, 22, 23]. On the other hand, climate change also leads to slow-onset and long-term environmental changes, such as droughts, desertification, or salinization. This latter type of environmental condition develops over a longer time horizon and typically allows individuals to adapt at least partially [24–27]. We contend that individuals’ exposure to these different manifestations of climate change, i.e., sudden- vs. gradual climate events, determines whether migration decisions are rooted in environmental reasons or rather economic concerns.

Eventually, we expect individuals to be strongly affected by the rapid and intense character of sudden-onset climate events. These occurrences tend to increase the salience of environmental conditions independent of people’s socio-economic status or adaptive capacity implying hardship for all affected individuals [5, 18, 28, 29]. As a result, respondents who were exposed to sudden-onset climate events should be more likely to have considered these events as the trigger for their migration decision–and, thus, will perceive themselves as environmental migrants. This theoretical expectation is supported by evidence that affected individuals’ tendency to recollect the intensity of such extreme events in a relatively consistent manner [30, 31], stressing the importance of taking into account people’s perceptions of climate events as they tend to affect their migration behavior [18, 21].

In contrast, in the case of slow-onset changes, any decision to move should only materialize after a longer time horizon during which individuals have tried to (unsuccessfully) adapt to changing environmental conditions [32]. Hence, people with different adaptive capacities and socio-economic statuses tend to perceive the same climate event differently [33]. The connection between climatic changes and the migration decision is then likely to be blurred by individuals’ assessment of their (failed) adaptation attempts, which increases the salience of other factors driving migration decisions, especially economic concerns. For slow-onset events, we correspondlingly do not expect individuals to self-classify as environmental migrants, even if these climatic changes might have triggered the relocation process in the first place. Note that while we explore environmental migrants and individuals’ self-identification as environmental migrants, our empirical identification strategy rests on asking people about their main reasons for migration. If they themselves name environmental reasons as one of their main reasons, we treat these individuals as environmental migrants. Methodologically, this approach avoids any social desirability biases in the survey, although it may induce a slight discrepancy between the concept of self-identification as an environmental migrant and listing environmental reasons as a cause for one’s migration decision.

The following sections describe our data and empirical strategy, before presenting the results. In the final section, we discuss the implications of our findings and conclude with an overview of the policy implications as well as avenues for future research stemming from our analysis.

## Materials and methods

We leverage original survey data on migrants from Vietnam and Kenya to test our theoretical expectations. We focus on these countries as they are among those most vulnerable to climate change according to the Global Climate Risk Index [34]. In both states, we surveyed migrants in three urban centers relying on a combination of referral and snowball sampling to identify and recruit participants. Only individuals of at least 16 years of age, who had lived in a rural area before moving to one of our survey sites (i.e., an urban center), and where they have stayed or intended to stay for at least six months were eligible to participate in our survey. Our convenience samples include 2,400 and 2,417 migrants in Vietnam and Kenya, respectively. Detailed information about survey sampling and implementation can be found in S1 Text in the Supporting Information.

The migrant is our unit of analysis. Given our interest in different types of migrants and, particularly, whether individuals classify themselves as “environmental migrants,” having migrants as the unit of analysis is the obvious choice when pursuing an efficient and effective analysis of the data. However, although this unit of analysis is suitable for our analysis, an alternative approach may be in need when exploring more general issues in the environment-migration nexus. For example, research on immobility or those studies interested in the distinction between migrants and non-migrants may need to apply a broader focus and include individuals as well who have not left their homes. Koubi et al. [32], for instance, use representative samples of non-migrant residents and referral samples of migrants to this end. Having said that, as our research focuses on migrants and their different motivations to leave, the migrant as such is the best unit of analysis for our purposes.

Our dependent variable, *Motive*, is a respondent’s primary reason(s) for migrating to an urban center. We asked respondents to name the three most crucial factors for why they “decided to leave their home location.” In total, individuals were shown a list of 25 reasons to choose from, which we aggregated to four main categories: social (e.g., no family/friends/relatives in previous location), political (e.g., persecution in previous location due to ethnic/religious/political beliefs), economic (e.g., no income, unemployment in previous location), and environmental reasons (e.g., the occurrence of flood, storm, drought, water/soil salinity). Additionally, we invited respondents to specify other reasons in an open-ended question, which we hand-coded and assigned to one of the aforementioned four aggregate categories. Ambiguous responses are coded as missing. In Kenya, 161 respondents submitted answers using the open-ended text field, and we were able to mannually assign about 50% of these (83 answers) to one of our migration-motive categories. The remaining answers were coded as missing. In Vietnam, we reassigned 33 (of 51) answers submitted in the open-ended text field.

As we are primarily interested in the conditions under which a migrant considers environmental factors as the key driver behind their migration decision, we merged all non-environmental reasons into one category and contrast these responses to respondents who selected an environmental motive among their top three relocation reasons. Accordingly, *Motive* is a binary item that assumes a value of 1 if a respondent cited an environmental reason for their migration (0 otherwise). Our data suggest that in both countries, a considerably larger share of migrants does not cite an environmental reason for their migration decision. In Kenya, only 38% of the respondents selected an environmental motive, while 62% reported that their decision to migrate was motivated by other influences. In Vietnam, almost 82% of the respondents cited exclusively non-environmental factors as the main factor for their move, more than four times the share of environmental migrants (18.4%).

Our main independent variable, *Disaster type*, captures a respondent’s objective exposure to climate events. We created this item using information from the geo-coded Emergency Events Database (EM-DAT) [35, 36]. We first matched the geo-coded location of occurred climatic and geographical conditions in EM-DAT to respondents’ geo-coded location of origin in the year they migrated. To this end, we initially geo-coded the description of respondents’ home locations using Google’s Geocoding Web Service. To identify this in the Vietnamese sample, respondents were asked to provide the name of the province, district, town, and commune where their previous home was located in. In Kenya, we used information about the province, state, town, and commune of the respondents’ former home. Note that 29 locations could not be geo-coded as the information provided in the survey suggest geocodes located outside of Kenya. We code these locations as missing. In Vietnam, all 2,400 survey responses were successfully geo-coded. Locations that recorded the occurrence of one or more floods, landslides, or storms were coded as respondents having been exposed to “sudden-onset” climatic events. Locations in which one or more droughts occurred in the year of respondents’ relocations were coded as having been exposed to “slow-onset” climate events. The reference category left out for comparison is linked to locations where no climate event(s) occurred.

The threshold criteria for including events in EM-DAT lead to the underreporting of smaller-scale events. Specifically, for disasters to be included in EM-DAT, they need to fulfil at least one of the following criteria: cause 10 or more deaths, inflict damage to 100 or more people, and trigger the declaration of a state emergency or a call for international assistance. Moreover, the EM-DAT administers, the Center for Research on the Epidemiology of Disasters, themselves caution that some disasters may go unreported as their coding relies mainly on disaster reporting from United Nations agencies, national governments, and the International Federation of Red Cross and Red Crescent Societies, which do not cover all disasters or have political limitations in reporting certain events. Thus, using EM-DAT may generate a more conservative measure of individuals’ exposure to climate events.

Our set of control variables includes both individual socio-demographic characteristics as well as objective climatic and geographical conditions in respondents’ home locations. To a large degree, these control items are meant to capture individuals’ adaptive capacity. People with high adaptive capacity should be less likely to identify themselves as environmental migrants. First, *Income* captures a respondent’s monthly income in the previous (pre-migration) location, including all formal and informal sources. It is measured on a 1–5 scale, with higher values indicating a higher income class. Second, we measure educational attainment (*Education*) with an ordinal variable on a 1–7 scale where 1 signifies a respondent has no formal education and 7 indicates completion of a postgraduate degree. Third, we compute a person’s age by deducting their year of birth from the year 2019, i.e., the year the survey was conducted in. We also include the squared *Age* term to model a non-linear impact on the environmental-migrant status. Fourth, there is a gender variable, with female respondents coded as 1 and male respondents as 0. Fifth, to measure respondents’ ownership of immobile assets in their previous location, we asked whether they owned properties such as houses or (farm) land. *Property* is a binary measure, coded as 1 if a respondent owned any of these assets before migrating (0 otherwise).

Finally, we include fixed effects for people’s ethnic group. To this end, we identified the following major factions in Kenya: Kikuyu, Luhya, Luo, Kamba, and Mijikenda. In Vietnam, over 90% of the respondents reported they belong to the Kinh, the largest ethnic group. This mirrors the latest Census data from 2019 [37]. We use binary variables to indicate whether a respondent belongs to any of these groups, with all ethnic groups that were selected by fewer than 100 respondents and non-Kinh respondents in Vietnam combined into one generic “other” category. We include survey question wording of all variables used in the main analysis in S1 Table of the Supporting Information.

Regarding objective climatic and geographical conditions in respondents’ home locations, we first control for the Standardized Precipitation Evapotranspiration Index (*SPEI*). Negative SPEI values stand for “drier-than-average conditions,” positive values indicate “wetter-than-average conditions” [38, 39]. For example, values ≥ +1.00 or ≤ −1.0 can be considered significant wet and dry periods, respectively. As we are mainly interested in absolute extreme conditions, we use the absolute values of the SPEI as our final variable.

We also include agro-ecological zones (AEZs) as an indicator of a location’s ability to support rainfed agriculture. An AEZ shares similar climatic conditions, including temperature, seasonality, and rainfall amounts and distribution, given the region’s latitude and elevation. To determine the AEZs of Kenya, we follow the classification by Sombroek et al. [40], which divides Kenya into seven zones ranging from humid to very arid. In Vietnam, AEZs overlap with the country’s geographical regions without a clear level of aridity associated with these regions. Following this classification, there are seven AEZs in Kenya and nine AEZs in Vietnam: North West (NW), Central Region and Northern Mountains (CRNM), North East (NE), Red River Delta (RRD), North Central Coast (NCC), South Central Coast (SCC), Central Highland (CH), Northeast of Southland (NES), and Mekong River Delta (MRD).

Moreover, we include groundwater access (*Groundwater*) using information from de Graaf et al. [41, 42]. This continuous variable indicates the average (logged) water table depth in meters at a respondent’s home location in the year of their reported migration.

Finally, we consider the logged distance between the geo-coded home of a respondent before migration and the location where the survey was conducted, i.e., the place the respondent migrated to. *Distance* is computed using the haversine formula and is measured in kilometers in direct distance between two points on a sphere. This variable thus captures geographical proximity–an important pull factor leading to migration. Summary statistics of all variables used in the main analysis are presented in S2 Table of the Supporting Information.

## Results

Given the dichotomous nature of our dependent variable, we run logistic regression models combined with robust standard errors and binary variables (fixed effects) for ethnic groups and agro-ecological zones (see Materials and Methods). Table 1 displays our main results in the form of three estimations: Model 1 is based on the Kenyan sample only, Model 2 exclusively focuses on Vietnam, and Model 3 pools the two countries’ data. The table entries are coefficients, which allow for a direct reading in terms of statistical significance and the direction of an effect. Substantive quantities of interest are presented in subsequent graphs that plot predicted probabilities and first-difference estimates.

**Table 1:** Effect of environmental events on identifying as environmental migrant.

	Model 1	Model 2	Model 3
	(Kenya)	(Vietnam)	(Pooled)
Slow-onset	0.119	0.149	0.258*
	(0.191)	(0.262)	(0.142)
Sudden-onset	0.299**	0.327**	0.638***
	(0.127)	(0.141)	(0.076)
Age	0.007	0.011	0.045**
	(0.033)	(0.035)	(0.022)
Age^2^	0.000	-0.000	-0.001**
	(0.000)	(0.000)	(0.000)
Female	0.078	-0.066	0.012
	(0.098)	(0.120)	(0.071)
Income	-0.045	-0.013	-0.272***
	(0.120)	(0.053)	(0.043)
Education	-0.174***	0.022	-0.165***
	(0.033)	(0.057)	(0.026)
Property	0.208*	0.240*	0.351***
	(0.109)	(0.141)	(0.076)
Distance	-0.044**	0.272***	-0.075***
	(0.020)	(0.070)	(0.015)
SPEI	0.154	0.015	0.139**
	(0.108)	(0.101)	(0.065)
Groundwater	0.006	-0.017	0.015
	(0.042)	(0.065)	(0.026)
Constant	0.225	-3.245***	-0.692*
	(0.656)	(0.916)	(0.413)
Observations	2,239	2,165	4,484
Pseudolikelihood (log)	-1,340.923	-981.236	-2,564.449

Robust standard errors in parentheses; constant, fixed effects for ethnic groups, and binary items for agro-ecological zones included in Models 1 and 2, but omitted from presentation.

*** p<0.01

** p<0.05

* p<0.1.

The results suggest, in line with our theoretical expectations, that migrants exposed to sudden-onset climate events in the location of origin are significantly more likely to cite environmental factors as one of the main reasons behind their migration decision. The point estimate of sudden-onset events ranges in [0.299; 0.638] across Models 1–3 and is statistically significant at the 5% level at least. In contrast, the coefficient of slow-onset events is–although positively signed–statistically insignificant in Models 1–2 and only significant at the 10%-level in Model 3, making the effect essentially indistinguishable from no exposure to any environmental event.

**Fig 1:**
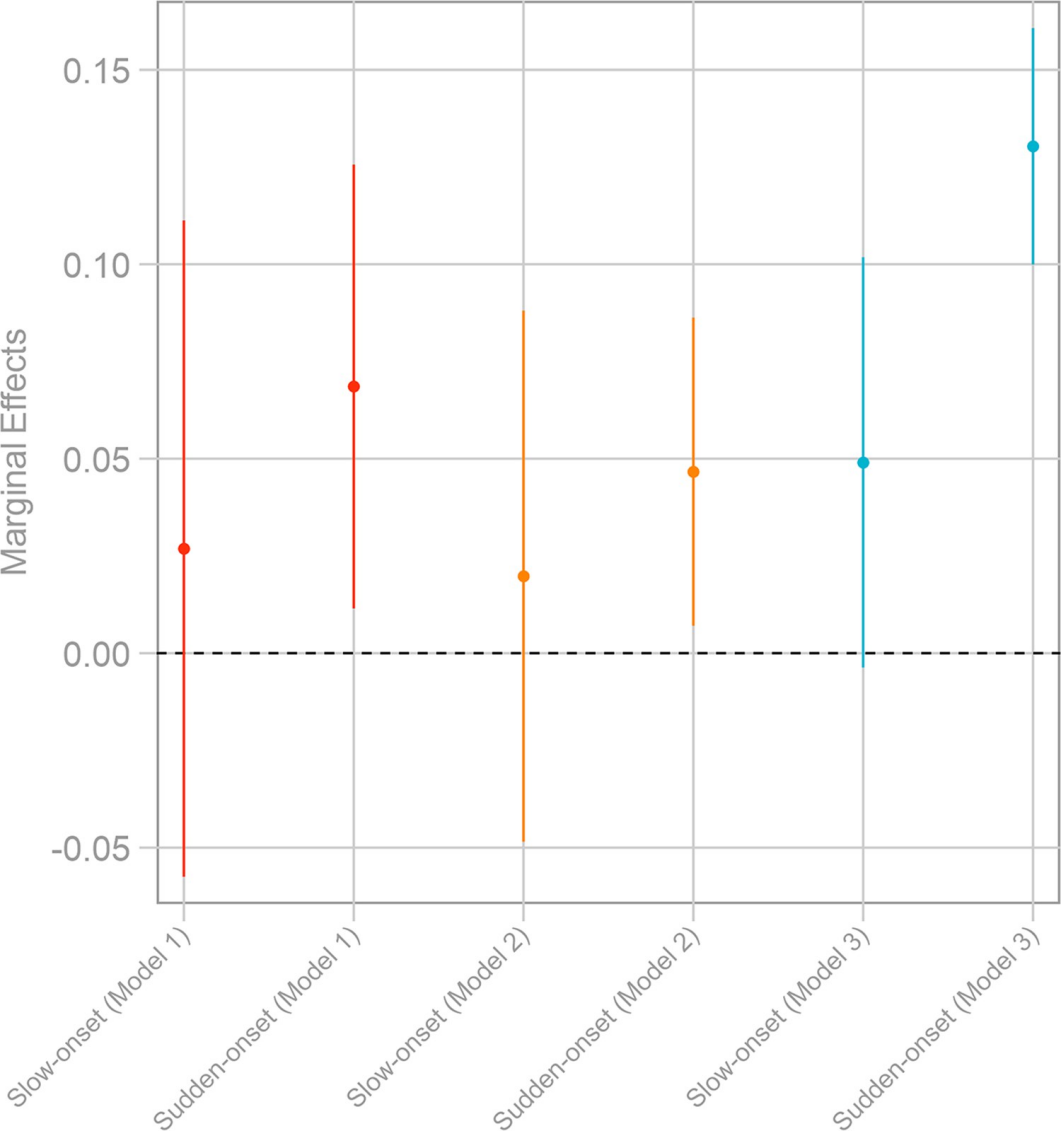
Marginal effects of environmental-events variables on environmental migrant. The graph displays marginal effects at the mean; calculations based on Table 1 while holding all other variables constant at their mean values; vertical bars signify 95 percent confidence intervals; marginal effect of 0 highlighted by the dashed horizontal line.

In Fig 1, we present the corresponding marginal effects for both slow- and sudden-onset environmental events while holding constant all other explanatory variables at their mean. The probability of perceiving oneself as an environmental migrant increases by about 5–7%-points when a migrant experienced a sudden-onset event in the previous location, i.e., before relocation. In the pooled sample, this effect increases to about 13%-points. The exposure to a slow-onset climatic event, in contrast, does not lead to a statistically different effect from the “no-exposure” scenario, as the confidence intervals cross the marginal-effect line of 0 in Fig 1 (despite the marginal effect point estimates being positive). Overall, these results, therefore, support our theoretical argument that experiencing sudden-onset climate events increases the salience of environmental changes as a critical driver of an individual’s decision to migrate.

The majority of respondents in our samples (81% in Vietnam and 68% in Kenya) say they have experienced at least one climate event in their previous location. However, despite having reported to have witnessed a climate event before, a large share of our respondents (78% in Vietnam and over 50% in Kenya) do not mention climatic changes as a driving factor behind their migration decisions. This further emphasizes the discrepancy between individuals’ exposure to climatic changes and the salience of environmental factors in their considerations to relocate. One explanation for this striking discrepancy might be that individuals who have experienced slow-onset climate changes are more likely to “underestimate,” i.e., wrongly classify these events compared to individuals exposed to sudden-onset events. To examine this possibility, we created a new outcome variable, *Disaster occurrence*, which captures respondents’ self-reporting of whether they have experienced a climate event in their previous location (coded as 1) or not (coded as 0). In turn, we regress this on people’s objective exposure to either a slow-onset or sudden-onset event. We rely on the same estimation procedure as in the previous models and expect that people who have experienced slow-onset climate events will be less likely to report exposure to an environmental change (although, objectively, this was the case).

**Fig 2:**
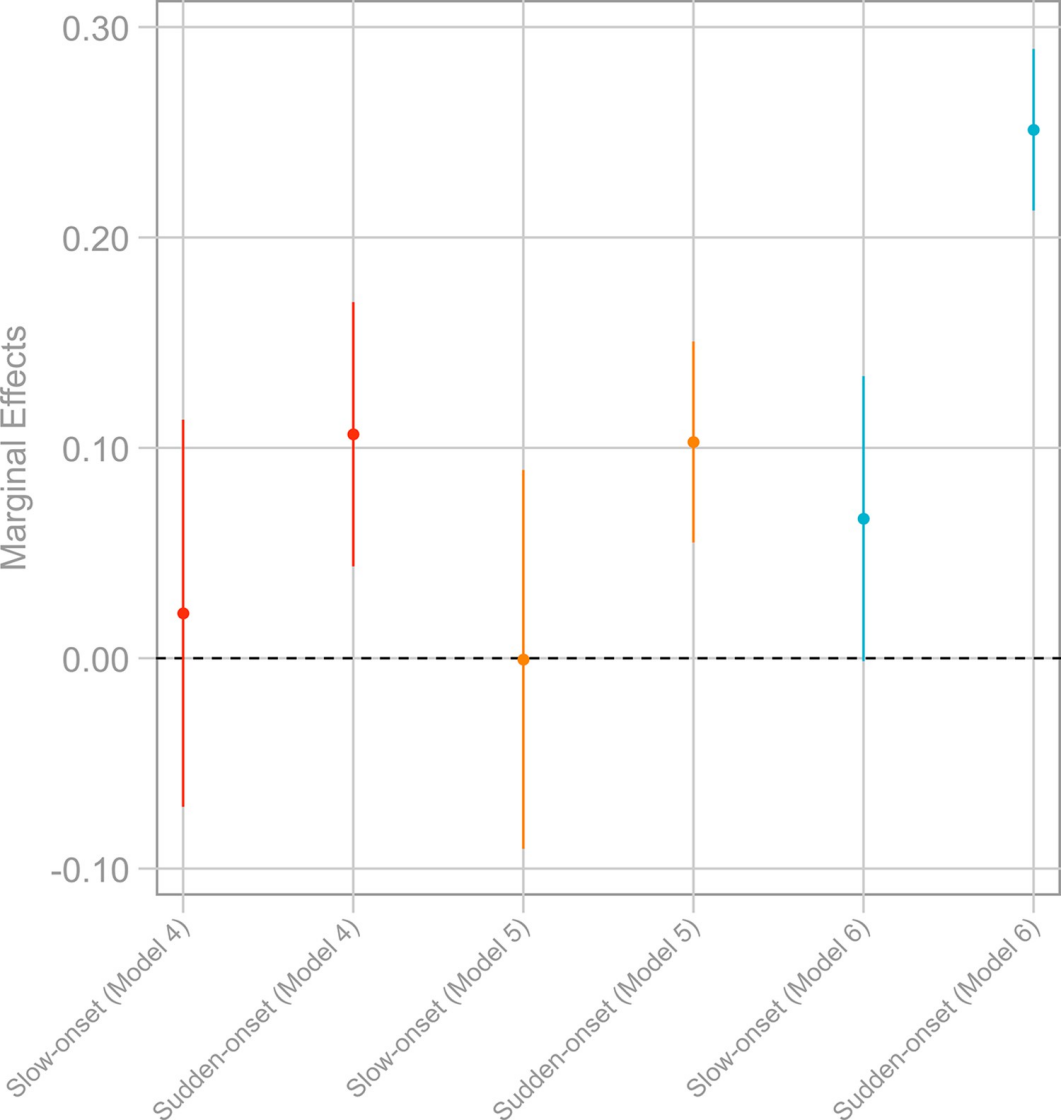
Marginal effects of environmental events variables on environmental disaster experience. The graph displays marginal effects at the mean; calculations based on Table 2 while holding all other variables constant at their mean values; vertical bars signify 95 percent confidence intervals; marginal effect of 0 highlighted by the dashed horizontal line.

The results summarized in Table 2 and Fig 2 support this claim and, thus, the explanation for the discrepancy between the number of migrants experiencing environmental events and those considering themselves as environmental migrants. All three models in Table 2 show that migrants from a location where sudden-onset climate events occurred before their relocation are more likely to report they had experienced environmental changes. In contrast, people exposed to long-term changes are not systematically more likely to do so. The marginal effects on the probability of saying that one experienced an environmental event before moving differ by more than 10%-points in Models 4–5 and almost by 30%-points in Model 6 if respondents have been exposed to a sudden-onset event according to our geo-coded data. In contrast, but as expected, the marginal effect estimates are all statistically insignificant for slow-onset environmental events. This indicates that migrants seem to underestimate, or have difficulties recollecting the severity of, slow-onset environmental changes as previously suggested by the literature [33].

**Table 2:** Effect of environmental events on environmental disaster experience.

	Model 4	Model 5	Model 6
	(Kenya)	(Vietnam)	(Pooled)
Slow-onset	0.097	-0.004	0.295*
	(0.212)	(0.290)	(0.153)
Sudden-onset	0.500***	0.810***	1.288***
	(0.150)	(0.191)	(0.104)
Age	-0.041	-0.076*	-0.048*
	(0.038)	(0.046)	(0.026)
Age^2^	0.001*	0.001*	0.001**
	(0.001)	(0.001)	(0.000)
Female	0.009	-0.000	-0.000
	(0.104)	(0.146)	(0.078)
Income	0.171	-0.159**	-0.027
	(0.132)	(0.068)	(0.049)
Education	-0.225***	0.137*	-0.123***
	(0.035)	(0.072)	(0.028)
Property	0.369***	0.042	0.005
	(0.111)	(0.191)	(0.085)
Distance	0.044*	0.344***	0.095***
	(0.023)	(0.106)	(0.017)
SPEI	-0.066	0.003	0.109
	(0.111)	(0.125)	(0.072)
Groundwater	-0.156***	-0.073	-0.187***
	(0.051)	(0.085)	(0.031)
Constant	3.806***	0.405	2.028***
	(0.981)	(1.164)	(0.484)
Observations	2,239	1,523	3,861
Pseudolikelihood (log)	-1,239.025		

Robust standard errors in parentheses; constant, fixed effects for ethnic groups, and binary items for agro-ecological zones included in Models 4 and 5, but omitted from presentation.

*** p<0.01

** p<0.05

* p<0.1.

**Fig 3:**
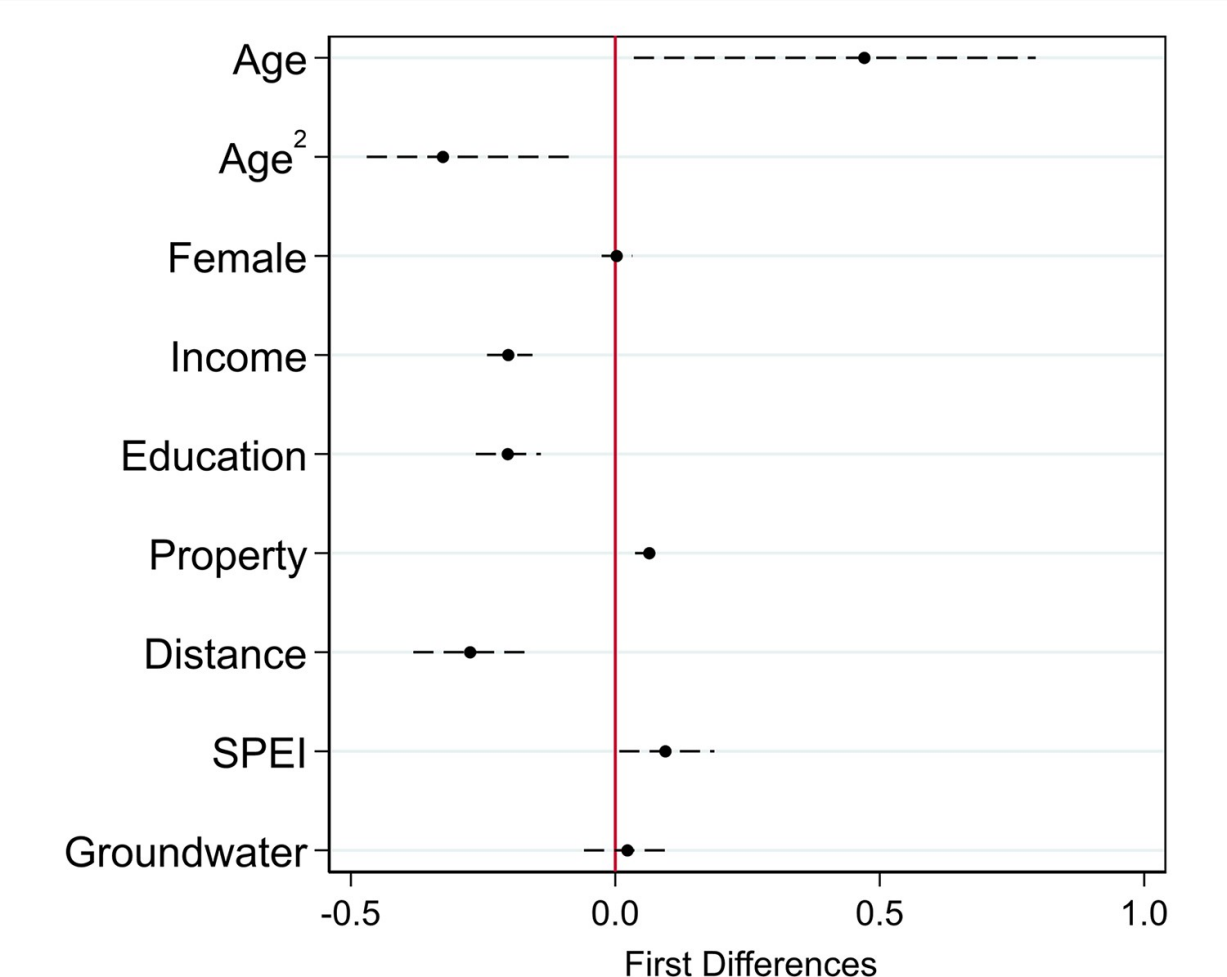
First difference estimates of the control variables. Calculations based on Model 3; vertical dashed bars signifiy 95 percent confidence intervals; first difference estimate of 0 marked with vertical solid line.

Regarding the control variables, Fig 3 plots the simulated first differences for each item (i.e., the change in the predicted probability of perceiving oneself as an environmental migrant when moving from the minimum to the maximum for each item) based on Model 3 in Table 1. The effect of the age-related variables provides some evidence for an inverted-U-shaped impact: individuals tend to identify themselves as environmental migrants with increasing age, but less likely so once a tipping point has been reached. According to Model 3, the tipping point is at 33 years of age. *Income*, *Education*, *Property*, and *Distance* are also significantly linked to respondents’ self-classification as environmental migration. According to our estimations, the higher a person’s income and education, the lower the likelihood of identifying oneself as an environmental migrant. The first-difference point estimates are -0.20 for *Income* and *Education*. *Property* is linked to a positive and statistically significant first difference, albeit the effect is relatively small (merely 0.06). Finally, the first difference estimate for *Distance* is -0.28. One reason for the latter effect being negatively associated with self-identification as an environmental migrant might be that it correlates with the type of events: sudden-onset events tend to lead to more mobility, but over shorter distance [43].

While we include dichotomous variables for ethnic groups and agro-ecological zones in the estimation of Models 1–2 and 4–5, their coefficients are omitted from the presentation to facilitate readability of the tables. However, there are some interesting results pertaining to these binary control variables nonetheless. First, for Kenya, migrants from the Luhya and Luo ethnicities are particularly more likely to classify themselves as environmental migrants. The Luo and Kamba people, in turn, are also more likely to have perceived environmental disasters in their previous home. In terms of the AEZs, the Central Region and Northern Mountains, North Central Coast, and Northeast of Southland zones are especially less prone to comprise people who not only see themselves as environmental migrants, but also who have perceived a climate disaster in their previous location. Second, for Vietnam, the only significant difference in terms of ethnic groups is given for Table 2: *Disaster occurrence*. The Kinh respondents were significantly less likely than others to have experienced an environmental disaster in their former homes. Coming to the Vietnamese AEZs, there are positive effects for the North Central Coast and South Central Coast zones: in both zones, respondents were more likely to see themselves as environmental migrants and to self-report that they have experienced a climate event in their previous location.

In the Supporting Information, we provide several robustness tests. First, we re-run our main analysis with a more fine-grained operationalization of our independent variable, *Disaster type*, to additionally capture respondents who have experienced both sudden-onset and slow-onset events (S3 and S4 Tables). The estimated effect of sudden-onset events on respondents’ likelihood to select environmental factors as a key motive of their migration as well as their likelihood to recall they have experienced an environmental disaster remains unchanged. However, the coefficients for slow-onset events are statistically insignificant in the individual country samples (Models S1, S2 and S4, S5), but positively signed and statistically significant effect for long-term events in the pooled samples (Models S3 and S6). With regard to the effect of experiencing both short-term and long-term event(s) the regression results suggest no consistent systematic effect on the likelihood to self-identify as an environmental migrant. However, these results should be interpreted with caution. There is only an extremely small portion of respondents in our country samples (<1%) who have migrated from locations that were affected by both types of environmental events. Thus, the effect size may have resulted from the test being statistically underpowered.

Second, we also account for the possibility that individuals’ decisions to migrate to a specific location may be driven by existing social networks at the migration destination, which can confound people’s motives for migration. To this end, we include the variable *Network* into the models, which is a binary item coded as 1 if a respondent confirms they have had core family members, relatives, former neighbors, or friends living at the place of destination before they migrated there (0 otherwise). Moreover, we consider respondents’ beliefs about the nature of climate change as a control variable. *Climate belief* is an ordinally scaled item: higher values indicate stronger beliefs in the seriousness of climate change as a problem and human responsibility. The results from the regression models, shown in S5 and S6 Tables, indicate that our main findings are robust to these changes in the model specifications.

## Discussion

Our empirical results allow for a number of clearly defined conclusions: while migrants who were exposed to sudden-onset event(s) in the location of origin are significantly more likely to cite environmental factors as one of the main reasons of their migration decision, migrants who were exposed to gradual-onset event(s) are not. The rationale behind this pattern is as follows. Sudden-onset events are often severe enough to trigger an immediate migration response as “people must flee from a rapid-onset environmental event to save their lives” [28: 405]. This typically implies that people have few possibilities to adapt when such types of environmental events hit. As a consequence, sudden-onset events tend to be perceived similarly by different individuals [33], and independent of their adaptive capacity or their socio-economic status affected individuals likely recollect the intensity of such events in a rather coherent manner [30, 31]. Individuals who have been exposed to sudden-onset events should then be more likely to think of themselves as environmental migrants.

When having been exposed to gradual-onset events, on the other hand, affected individuals might not perceive the underlying climatic event, e.g., the drought, to be the main driver of their final migration decision. Instead, they rather “blame” the economic hardship that followed from the altered environment for their migration decision [44]. Eventually, there is no increased likelihood of naming environmental reasons as one of the main causes for affected individuals’ migration decision. This argument is further corroborated by our second set of results, which show that individuals find it more difficult or rather show more variation in recollecting these gradual-onset events in contrast to when they have experienced sudden-onset events.

One caveat concerning our analysis is that the size of the effect of experiencing a sudden-onset event on the likelihood of self-identifying as an environmental migrant, though statistically significant, is less substantively important. While we do not make an argument concerning the size of the effect, one could claim that a 13%-point increase is not very large in light of the scale from 0 to 100 percent. This might be driven by various reasons, and we know that migration decisions probably are throughout multidimensional [2, 3], but this effect could well be more strongly pronounced when disaggregating environmental events also by the degree of damage done and the number of fatalities caused. For instance, more intensive events causing a lot of infrastructure damage and casualties receive more media attention and, in turn, are more likely to be noticed by individuals [45]. Considering this, it may be an effort worth making to employ in future research a different or more extensive disaggregation of environmental events next to the one we offer here.

## Conclusion

Who perceives themselves as an environmental migrant? We contend that the experience of climate-induced environmental changes does not necessarily lead to individuals’ self-identification as environmental migrants. Instead, the type of climate event should at least partially determine whether people consider environmental changes as the main reason for moving locations. We have put forward the argument that sudden-onset climate events affect most people equally and, because of their immense destructive power, tend to lead to a mainly uniform migratory response: individuals who have been experiencing such events should be more likely to think of themselves as environmental migrants. On the contrary, when experiencing slow-onset climatic changes, individuals’ capacity to adapt should matter more for both their perception of climatic change as such and whether they connect corresponding environmental changes to their decision to migrate. Furthermore, many people who become migrants after having experienced slow-onset climate events only do so after more time elapsed in which they have tried to adapt *in situ*. Hence, we claim that, in this case, the main reason for migrating for many people likely will be an economic one–instead of underlying environmental changes.

The findings based on original survey data from Kenya and Vietnam, two countries highly vulnerable to climate change, support our theory. Individuals who experienced sudden-onset climate events are more likely in turn to self-identify as environmental migrants than those who experienced slow-onset climatic changes. These results seem to corroborate evidence from macro-level studies, which indicate that sudden-onset events tend to cause more sudden and involuntary migrations, while slow-onset events result in immobility or produce movements that are generally perceived as being voluntary and often predominantly economically motivated [4, 5].

We consider our study to be relevant for the broader academic literature on climate change and migration as well as for policymakers. Since our approach combines both actual climatic data with individual-level data on the perception of environmental changes, it contributes to the nascent literature seeking to investigate how climate change maps onto the real life of affected people. Furthermore, a better understanding of when and why people “blame” the environment instead of the economy or the political/social circumstances when deciding to migrate could have important implications for security considerations: for example, individuals might become more aggrieved when misattributing the real causes of migration. In particular, if migrants believe that they had to move because of economic reasons, although it was climate change that led to migration, they could attribute blame on their government fueling grievances inducing greater frustration vis-à-vis the state; ultimately, the risk of protests and domestic-level conflict could rise [46]. Understanding such dynamics to potentially avoid their worst consequences seems highly relevant.

In addition, our research has implications for the debate on establishing an environmental/climate refugee status. While currently no clear legal status of “climate refugees” exists, present international developments make it likely that climate refugees will be treated similarly to (political) refugees in the future and, consequently, legal protection afforded to refugees could be extended to them [47]. In light of our findings, this implies that (environmental) migrants who flee sudden-onset climate events will be likely to claim such status. However, this could lead to the exclusion of people migrating due to slow-onset events who will either not link their relocation to environmental changes in the first place because they did not perceive these as the root cause of their migration decision; or they will find it more difficult showing that gradual-onset events indeed played a major role in their migration decisions, since these occurences tend to work more indirectly via their adverse effect on people’s economic well-being [48]. Consequently, reducing the issue of migration in the context of climate change to the status of “environmental refugees” fails to recognize that environmental/climatic factors are rarely the single reason for migration, leading to partial solutions to address the complex climate change-migration relationship.

## Supporting information

S1 Text Survey sampling and implementation. (PDF)

S1 Table Question wording of key variables. (PDF)

S2 Table Descriptive statistics of key variables. (PDF)

S3 Table Effect of environmental events on identifying as environmental migrant: Including both slow-onset and sudden-onset events. (PDF)

S4 Table Effect of environmental events on environmental disaster experience: Including both slow-onset and sudden-onset events. (PDF)

S5 Table Estimated effect of type of environmental events on likelihood to identify as environmental migrant. (PDF)

S6 Table Estimated effect of type of environmental events on likelihood to report having experienced an environmental disaster. (PDF)
