# Supplementary material for: How sudden- versus slow-onset environmental events affect self-identification as an environmental migrant: Evidence from Vietnamese and Kenyan survey data
Source: PLoS One. 2024 Jan 25;19(1):e0297079. doi: 10.1371/journal.pone.0297079 (PMC10810492; doi:10.1371/journal.pone.0297079)
Supplement: S1 Text — (PDF) [file pone.0297079.s001.pdf]

S1 Text. Survey sampling and implementation

The selection of Kenya and Vietnam as our study countries was guided by their high vulnerability to climate change as indicated by multiple rankings, including the Global Climate Risk Index. This deliberate choice increases the likelihood of identifying regions with significant exposure to environmental, climate-induced disasters within these countries. For each selected country, we opted for a three-site approach to ensure geographical diversity, focusing on cities known for attracting high numbers of internal migrants. While the initial city selection was not specifically based on their exposure levels to sudden and slow-onset events, our analysis reveals notable variations in the extent to which these countries have experienced different types of natural disasters.

On average, both Vietnam and Kenya exhibit a higher share of exposure to sudden-onset events, with a rate of 36% and 22%, respectively. Nevertheless, sub-national variations are apparent. For instance, Mombasa in Kenya experienced 91 sudden-onset events and 122 slow-onset events, illustrating a diverse natural-disaster profile. In contrast, Nairobi faced 171 sudden-onset events and 41 slow-onset events, which signifies a different pattern of exposure. These insights underscore the complexity and diversity of natural-disaster experiences within the chosen countries.

In each country, we surveyed about 2,400 migrants from three cities: Hanoi, Ho Chi Minh City, and Binh Duong in Vietnam; and Nairobi, Mombasa, and Kisumu in Kenya.

A respondent is categorized as a migrant if they meet the following conditions:

- They are between 16 and 65 years old.
- They were born in a rural area.
- They relocated to the surveyed city at the age of 16 or later.
- They have either been residing in or plan to stay in the surveyed city for a duration of at least 6 months.
- Prior to moving to the surveyed city, they had lived in a rural area for a period of at least 1 year.

Due to a lack of official data (e.g., household lists) of migrant populations from local authorities,[1] we were unable to generate a sampling frame from which to systematically draw random samples. We, therefore, relied on a combination of convenience sampling and snowballing methods.

The sampling process consisted of four stages. Initially, in each city, a random selection was made of 3-4 districts (Vietnam)/constituencies (Kenya) from the two largest cities and a smaller one. The chosen districts/constituencies were as follows: Hanoi (Dong Anh District, Me Linh District, Nam Tu Liem District, Gia Lam District); Binh Duong (Thu Dau Mot City, Ben Cat Town, Di An Town, Thuan An Town); HCMC (Binh Chanh District-Tan Phu District, Thu Duc District, Go Vap District); Nairobi (Dagoretti North, Embakasi, Kasarani, Ruaraka);

[1] For example, the list of migrants in Hanoi surveyed in the 2015 National Domestic Migration Survey provided by General Statistics Office and the list of laborers from the 2017 Labor Force Survey provided by Vietnam's Ministry of Labor, Invalids and Social Affairs (MOLISA) do not contain the information we need: the previous location before migration and the time of migration.

Mombasa (Jomvu, Kisauni, Likoni, Mvita); Kisumu (Kisumu Central, Kisumu East, Kisumu West).

Next, within each district/constituency, ten enumeration sites were randomly chosen. At each enumeration site, a starting point was identified from which enumerators would commence the selection of households. Typically, starting points were in central areas of the district/constituency, such as the central market, a hospital, or a local community building.

Lastly, for household selection, interviewers followed the right-hand rule, skipping every second house starting from the designated starting point. Additionally, an effort was made to ensure gender and age balance by implementing quotas to achieve a roughly equal number of respondents from each group.

The surveys were conducted face-to-face using tablets in January/February 2019. On average, respondents took 40-50 minutes to complete the questionnaire.
