# Supplementary material for: How sudden- versus slow-onset environmental events affect self-identification as an environmental migrant: Evidence from Vietnamese and Kenyan survey data
Source: PLoS One. 2024 Jan 25;19(1):e0297079. doi: 10.1371/journal.pone.0297079 (PMC10810492; doi:10.1371/journal.pone.0297079)
Supplement: S1 Table — (PDF) [file pone.0297079.s002.pdf]

S2 Table. Question wording of key variables

Variable	Question	Response categories
Migration motive	What would you say are the most important reason(s) you decided to leave your home location. Please list up to three reasons.	*Social/Political*<br>• No family/relatives/friends in former location<br>• Inadequate schooling in former location]<br>• Inadequate health care in former location]<br>• Lack of social/ cultural/ recreational opportunities in previous location<br>• Discrimination in previous location<br>• Insecurity [physical/sexual] in previous location<br>• Exploitation [labor/sexual] in previous location<br>• Familial conflict in previous location<br>• Enmity at village<br>• Political conflict in previous location<br>• Persecution in previous location due to ethnic/religious/ political beliefs<br>• Discrimination in previous location due to ethnic/ religious/ political beliefs<br>• Harassment in previous location due to ethnic/ religious/ political beliefs<br>*Economic*<br>• Not enough income<br>• Poor soil quality<br>• Poor water quality (contamination)<br>• No wood available<br>*Environmental*<br>• Water shortage/ Drought/ Water or Soil Salinity (1 event)<br>• Repeated droughts/Long term salinity<br>• Too much rain<br>• Short term events such as flood, storm, landslide, cyclone/hurricane<br>*Other*
Disaster occurrence	Have there been any environmental disasters in [] while you were living there?	0 No<br>1 Yes
Age	When were you born?	---
Ethnicity	Which ethnic group do you belong to?	---
Income	What was your average individual monthly income at your **previous location**, including formal and informal sources?	1 Less than 1 million VND/ Less Than 10,000 Shilling<br>2 1-3 million VND/ 10,001 – 30,000 Shilling<br>3 3-5 million VND/ 30,001 – 50,000 Shilling<br>4 5-8 million VND/ 50,001 – 100,000 Shilling<br>5 8-12 million VND/ Over 100,000 Shilling<br>6 More than 12 million VND
Education	What is the highest level of education you have attended?	1 Never went to school<br>2 Some primary education<br>3 Completed primary education<br>4 Completed lower secondary education<br>5 Completed upper secondary education<br>6 University degree<br>7 Postgraduate degree
Property (Index)	For each of the listed items, please tell us whether you owned the item at your **previous location**?<br>- House or apartment<br>- Farm<br>- Piece of land or garden for subsistence<br>- Animals for subsistence<br>- Shop/factory	0 No<br>1 Yes
Network	Before you arrived in [], did you have core family members, relatives, former neighbors or friends already living here?	0 No<br>1 Yes
Climate belief	Thinking about climate change, also called global warming, which of the following statements best describes your opinion?	1 Climate change is not happening.<br>2 Climate change is entirely caused by natural processes.<br>3 Climate change is partly caused by natural processes and partly caused by human activity.<br>4 Climate change is mainly caused by human activity.<br>5 Climate change is completely caused by human activity.
