# Supplementary material for: How sudden- versus slow-onset environmental events affect self-identification as an environmental migrant: Evidence from Vietnamese and Kenyan survey data
Source: PLoS One. 2024 Jan 25;19(1):e0297079. doi: 10.1371/journal.pone.0297079 (PMC10810492; doi:10.1371/journal.pone.0297079)
Supplement: S2 Table — (PDF) [file pone.0297079.s003.pdf]

S3 Table. Descriptive statistics of key variables

	KENYA					VIETNAM				
	N	Mean	SD	Min	Max	N	Mean	SD	Min	Max
Migration motive	2,416	0.381	0.486	0	1	2,400	0.184	0.387	0	1
Disaster occurrence	2,416	0.676	0.468	0	1	1,713	0.809	0.393	0	1
Disaster type	2,416	1.497	0.825	1	3	2,400	1.735	0.937	1	3
Age	2,416	29.34	8.515	18	65	2,400	29.71	10.41	17	66
Ethnicity	2,405	2.681	1.474	0	5	2,400	0.905	0.293	0	1
Income	2,352	1.166	0.432	1	5	2,170	2.014	1.138	1	6
Education	2,379	4.389	1.525	1	7	2,399	4.784	1.268	1	7
Female	2,416	0.537	0.499	0	1	2,400	0.540	0.498	0	1
Property	2,416	0.690	0.463	0	1	2,400	0.352	0.478	0	1
Distance	2,416	4.269	2.971	-9.216	6.841	2,400	5.291	0.973	1.503	7.142
SPEI	2,416	1.242	0.446	0.00285	3.011	2,400	1.279	0.609	0.0132	3.355
Groundwater	2,416	3.739	1.525	-0.316	7.138	2,400	2.983	1.223	0.946	6.790
Network	2,416	0.755	0.430	0	1	2,400	0.607	0.489	0	1
Climate belief	2,274	3.069	0.940	1	5	2,339	3.345	0.992	1	5
AEZs	2,348	3.937	1.398	1	7	2,396	4.804	2.496	1	9
