# Supplementary material for: How sudden- versus slow-onset environmental events affect self-identification as an environmental migrant: Evidence from Vietnamese and Kenyan survey data
Source: PLoS One. 2024 Jan 25;19(1):e0297079. doi: 10.1371/journal.pone.0297079 (PMC10810492; doi:10.1371/journal.pone.0297079)
Supplement: S3 Table — (PDF) [file pone.0297079.s004.pdf]

S4 Table. Effect of environmental events on identifying as environmental migrant: Including both slow-onset and sudden-onset events

	Model S1 (Kenya)	Model S2 (Vietnam)	Model S3 (Pooled)
Slow-onset	0.087	0.303	0.340**
	(0.194)	(0.294)	(0.150)
Sudden-onset	0.303**	0.329**	0.637***
	(0.127)	(0.141)	(0.076)
Both	1.329	-0.344	-0.342
	(1.143)	(0.553)	(0.426)
Age	0.007	0.011	0.044**
	(0.033)	(0.035)	(0.022)
Age^2	0.000	-0.000	-0.001**
	(0.000)	(0.000)	(0.000)
Female	0.078	-0.067	0.013
	(0.098)	(0.120)	(0.071)
Income	-0.050	-0.015	-0.269***
	(0.120)	(0.053)	(0.043)
Education	-0.174***	0.017	-0.166***
	(0.033)	(0.057)	(0.026)
Property	0.212*	0.241*	0.351***
	(0.109)	(0.141)	(0.076)
Distance	-0.044**	0.269***	-0.074***
	(0.020)	(0.070)	(0.015)
SPEI	0.157	0.011	0.137**
	(0.108)	(0.102)	(0.065)
Groundswater	0.004	-0.012	0.014
	(0.042)	(0.065)	(0.026)
Constant	0.162	-3.227***	-0.674
	(0.659)	(0.916)	(0.413)
Observations	2,239	2,165	4,484

Robust standard errors in parentheses; constant, fixed effects for ethnic groups, and binary items for agro-ecological zones included in Models S1 and S2, but omitted from presentation.

*** $p<0.01$, ** $p<0.05$, * $p<0.1$.
