# Supplementary material for: How sudden- versus slow-onset environmental events affect self-identification as an environmental migrant: Evidence from Vietnamese and Kenyan survey data
Source: PLoS One. 2024 Jan 25;19(1):e0297079. doi: 10.1371/journal.pone.0297079 (PMC10810492; doi:10.1371/journal.pone.0297079)
Supplement: S4 Table — (PDF) [file pone.0297079.s005.pdf]

**S5 Table. Effect of environmental events on environmental disaster experience: Including both slow-onset and sudden-onset events**

| | Model S4 (Kenya) | Model S5 (Vietnam) | Model S6 (Pooled) |
|---|---|---|---|
| Slow-onset | 0.112 | -0.196 | 0.303* |
| | (0.214) | (0.348) | (0.162) |
| Sudden-onset | 0.499*** | 0.809*** | 1.288*** |
| | (0.150) | (0.191) | (0.104) |
| Both | -0.782 | 0.296 | 0.243 |
| | (0.918) | (0.472) | (0.420) |
| Age | -0.041 | -0.077* | -0.048* |
| | (0.038) | (0.046) | (0.026) |
| $Age^2$ | 0.001* | 0.001* | 0.001** |
| | (0.001) | (0.001) | (0.000) |
| Female | 0.009 | 0.003 | -0.000 |
| | (0.104) | (0.147) | (0.078) |
| Income | 0.171 | -0.160** | -0.027 |
| | (0.132) | (0.068) | (0.049) |
| Education | -0.226*** | 0.140* | -0.123*** |
| | (0.035) | (0.072) | (0.028) |
| Property | 0.369*** | 0.043 | 0.005 |
| | (0.111) | (0.191) | (0.085) |
| Distance | 0.044* | 0.345*** | 0.095*** |
| | (0.023) | (0.106) | (0.017) |
| SPEI | -0.067 | 0.011 | 0.109 |
| | (0.111) | (0.126) | (0.072) |
| Groundswater | -0.155*** | -0.079 | -0.187*** |
| | (0.051) | (0.085) | (0.031) |
| Constant | 3.887*** | 0.420 | 2.031*** |
| | (1.018) | (1.164) | (0.484) |
| Observations | 2,239 | 1,523 | 3,861 |

Robust standard errors in parentheses; constant, fixed effects for ethnic groups, and binary items for agro-ecological zones included in Models S4 and S5, but omitted from presentation.

*** p<0.01, ** p<0.05, * p<0.1.
