# Supplementary material for: How sudden- versus slow-onset environmental events affect self-identification as an environmental migrant: Evidence from Vietnamese and Kenyan survey data
Source: PLoS One. 2024 Jan 25;19(1):e0297079. doi: 10.1371/journal.pone.0297079 (PMC10810492; doi:10.1371/journal.pone.0297079)
Supplement: S5 Table — (PDF) [file pone.0297079.s006.pdf]

S6 Table. Estimated effect of type of environmental events on likelihood to identify as environmental migrant

	Model S7 (Kenya)	Model S8 (Vietnam)	Model S9 (Pooled)
Slow-onset	0.184	0.145	0.282**
	(0.195)	(0.263)	(0.143)
Sudden-onset	0.312**	0.289**	0.638***
	(0.131)	(0.142)	(0.078)
Age	0.023	0.015	0.054**
	(0.034)	(0.036)	(0.022)
Age^2	-0.000	-0.000	-0.001**
	(0.000)	(0.000)	(0.000)
Female	0.099	-0.057	0.033
	(0.101)	(0.121)	(0.072)
Income	-0.051	-0.019	-0.273***
	(0.125)	(0.054)	(0.044)
Education	-0.175***	0.022	-0.166***
	(0.035)	(0.059)	(0.027)
Property	0.178	0.240*	0.330***
	(0.112)	(0.143)	(0.078)
Network	-0.188*	0.041	0.092
	(0.110)	(0.119)	(0.075)
Climate belief	0.048	-0.022	0.006
	(0.052)	(0.062)	(0.036)
Distance	-0.040**	0.285***	-0.070***
	(0.020)	(0.070)	(0.016)
SPEI	0.151	0.025	0.125*
	(0.112)	(0.102)	(0.066)
Groundwater	0.006	-0.003	0.007
	(0.044)	(0.066)	(0.027)
Constant	-0.126	-3.375***	-0.926**
	(0.700)	(0.938)	(0.438)
Observations	2,107	2,110	4,294

Robust standard errors in parentheses; constant, fixed effects for ethnic groups, and binary items for agro-ecological zones included in Models S7 and S8, but omitted from presentation.

*** p<0.01, ** p<0.05, * p<0.1
