# Supplementary material for: How sudden- versus slow-onset environmental events affect self-identification as an environmental migrant: Evidence from Vietnamese and Kenyan survey data
Source: PLoS One. 2024 Jan 25;19(1):e0297079. doi: 10.1371/journal.pone.0297079 (PMC10810492; doi:10.1371/journal.pone.0297079)
Supplement: S6 Table — (PDF) [file pone.0297079.s007.pdf]

S7 Table. Estimated effect of type of environmental events on likelihood to report having experienced an environmental disaster

	Model S10 (Kenya)	Model S11 (Vietnam)	Model S12 (Pooled)
Slow-onset	0.105	0.075	0.292*
	(0.214)	(0.292)	(0.154)
Sudden-onset	0.564***	0.883***	1.343***
	(0.157)	(0.195)	(0.107)
Age	-0.037	-0.072	-0.040
	(0.039)	(0.048)	(0.028)
Age^2	0.001	0.001	0.001*
	(0.001)	(0.001)	(0.000)
Female	0.004	-0.045	0.011
	(0.106)	(0.151)	(0.080)
Income	0.193	-0.185***	-0.034
	(0.137)	(0.069)	(0.050)
Education	-0.231***	0.165**	-0.130***
	(0.036)	(0.078)	(0.030)
Network	0.122	0.502***	0.189**
	(0.114)	(0.146)	(0.084)
Climate belief	0.045	-0.002	0.099**
	(0.054)	(0.078)	(0.042)
Distance	0.041*	0.336***	0.095***
	(0.024)	(0.109)	(0.018)
SPEI	-0.064	0.055	0.112
	(0.113)	(0.130)	(0.074)
Groundwater	-0.151***	-0.087	-0.181***
	(0.052)	(0.088)	(0.032)
Constant	3.426***	-0.071	1.466***
	(1.012)	(1.215)	(0.523)
Observations	2,107	1,487	3,690

Robust standard errors in parentheses; constant, fixed effects for ethnic groups, and binary items for agro-ecological zones included in Models S10 and S11, but omitted from presentation.

*** $p<0.01$, ** $p<0.05$, * $p<0.1$.
